# Why is there still hepatitis C transmission in Australian prisons? A case report

**DOI:** 10.1186/s12954-017-0201-y

**Published:** 2017-11-25

**Authors:** Ben Harkness, Michael Levy, Ruth Evans, Jillian Wenke

**Affiliations:** 1 Justice Health Services, ACT Health, Canberra, Australia; 2 Justice Health Services, ACT Health, ANU Medical School, Canberra, Australia

**Keywords:** Hepatitis C, Prisoner health, Direct-acting antivirals, Re-infection, Needle syringe programme

## Abstract

**Background:**

The ability to cure hepatitis C viral infection, with specific reference to the prisoner population and the prison environment, will be challenged, even if opiate replacement therapy is concurrently offered, and even if bleach is available. The missing elements, widely available in the community, are a regulated injecting equipment exchange and tattooing parlours.

**Case presentation:**

We report a case of re-infection of hepatitis C in a prisoner treated with a direct-acting antiviral. What makes this case so remarkable is that it was entirely predictable and preventable.

**Conclusions:**

Hepatitis C infection will continue to test both the strengths and the weaknesses in the relationship between health and corrective services in Australia. Nothing less than full implementation of all harm minimisation modalities will be necessary to eliminate the clinical and public health risks of hepatitis C infection, both in prison and by extension into the general community.

## Background

The ability to cure hepatitis C viral (HCV) infection, with specific reference to the prisoner population and the prison environment, will be challenged, even if opiate replacement therapy is concurrently offered, and even if bleach is available. The missing elements, widely available in the community, are a regulated injecting equipment exchange and tattooing parlours.

We report a case of re-infection of hepatitis C in a prisoner treated with a direct-acting antiviral (DAA). What makes this case so remarkable is that it was entirely predictable [1] and preventable.

Treating prisoners infected with HCV is a priority of the Fourth Australian Hepatitis C Strategy. That document states:

‘The prevalence of hepatitis C is disproportionately higher among people in custodial settings, due primarily to a high rate of imprisonment for drug-related offences and unsafe injecting drug use in prisons’ [2].

The Australian Government’s position is strongly supported by public health principles [3, 4].

Reasons for this sensible approach are the risks of transmission in-custody of an already highly infected population, their chaotic lifestyle in the community, associated toxicity of interferon-based treatments and their need for supervision, high rates of serious mental illness and other comorbidities. Further, DAAs have been reported to be safe and cost-effective in the prison setting [5].

On 1 March 2016, the Australian Minister for Health authorised the prescription of DAAs to Australian citizens with proven chronic hepatitis C infection. The Australian Medical Association has stated that funding was linked to the ‘controversial axing of bulk billing incentives for pathology and diagnostic imaging services’ [6]. Remarkably, and responding to clear public health imperatives, Australian prisoners were fully entitled to access publicly funded DAAs—absolutely cost-free to patients.

Australia could be a world achiever, by this early adoption of universal access to DAA medications. In contrast, in the USA, prisoners have virtually no access to these medications [7].

## Case presentation

The 39-year-old male was treated for HCV genotype 1a between weeks 4 and 16 of incarceration (12 weeks of ledipasvir and sofosbuvir). Four weeks after the therapy was finished, the patient returned a negative polymerase chain reaction (PCR) result.

The patient describes a single episode of shared injecting with a known HCV-positive contact. The episode is described with some clarity, approximately 4–6 weeks after the negative PCR result. Bleach had been accessed, and a well-described method of flushing the injecting equipment with diluted bleach was used. The patient was receiving a stable dose of 95 mg methadone at the time.

He had been in custody for 7 months when the health service was notified that the HCV PCR result was positive. That result related to a test taken in week 30 of the period of incarceration. The genotype was reported as a mixed infection of genotype 3 and genotype 1 or 6—clearly distinct from the sample taken at pre-treatment. Given the significance of that result, the sample was retested and confirmed. Liver function tests and full blood count were normal.

## Discussion and conclusion

The Alexander Maconochie Centre (AMC) is presented by the Australian Capital Territory (ACT) Government as the first human rights-compliant prison in Australia. The ACT Government committed to the provision of sterile injecting equipment through a regulated needle and syringe programme (NSP) [8]. Regrettably, that announcement was premature—in 2016, custodial officers voted overwhelming to reject any proposed model, and the initiative has, to date, been buried. This NSP was to complement an opiate replacement programme and the confidential provision of bleach, both bulk liquid and in powder form packed in individual use sachets. Approximately one-third of prisoners are on methadone-maintenance therapy, including the patient in this case report.

Late relapse of HCV infection after DAA treatment is described [9], but we believe that we are describing an instance of re-infection, rather than relapse, for the following reasons:

- The patient completed 12 weeks of prescribed treatment with ledipasvir and sofosbuvir for genotype 1a HCV infection
- Was 100% compliant with dosing—confirmed against his medication charts
- Provides a credible history of injecting drug use between the two qualitative PCR tests (referred to as ‘end-of-treatment’ and ‘sustained viral-response’)
- The genotype change before and after treatment

Our patient was counselled that he was once again ‘infectious’ and that a decision to retreat would be made in 6 months’ time, only once chronic infection has been determined. Interestingly, pathology taken at 6 months revealed a negative PCR result, indicating the patient had spontaneously cleared his HCV re-infection.

We believe that this case report highlights a number of health and ethical issues:

- The decision to offer prisoners equal access to DAA treatments for HCV infection was well grounded, both epidemiologically and ethically [4]
- The risk of HCV transmission, while reduced by rapid implementation of DAA treatment, was not completely eliminated and cannot be expected to be until every PCR HCV-positive patient is rendered non-infectious
- There will be ongoing ‘transmission’ of HCV from community to prison, with the constant flow of new receptions into custody
- Bleach availability is, at best, partially effective in reducing HCV transmission
- Widespread prescription of opiate replacement treatment, similarly, is demonstrated to be at best, partially effective in reducing HCV transmission
- A regulated injecting equipment exchange would have provided a respectful and humane choice to our case and further reduced the risk of re-infection

Hepatitis C infection will continue to test both the strengths and the weaknesses in the relationship between health and corrective services in Australia. Nothing less than full implementation of all harm minimisation modalities will be necessary to eliminate the clinical and public health risks of HCV infection, both in prison and by extension into the general community.

## Abbreviations

ACT: Australian Capital Territory
AMC: Alexander Maconochie Centre
DAA: Direct-acting antiviral
HCV: Hepatitis C virus
NSP: Needle syringe programme
PCR: Polymerase chain reaction
